# In-flight allergic emergencies

**DOI:** 10.1186/s40413-017-0148-1

**Published:** 2017-05-04

**Authors:** Mario Sánchez-Borges, Victoria Cardona, Margitta Worm, Richard F. Lockey, Aziz Sheikh, Paul A. Greenberger, Ignacio J. Ansotegui, Motohiro Ebisawa, Yehia El-Gamal, Stanley Fineman, Mario Geller, Alexei Gonzalez-Estrada, Luciana Tanno, Bernard Y. Thong, on behalf of the WAO Anaphylaxis Committee

**Affiliations:** 1 Allergy and Clinical Immunology Department, Centro Médico Docente La Trinidad, Caracas, Venezuela; 2 Allergy Section, Department of Internal Medicine, Hospital Universitari Vall d’Hebron, Barcelona, Spain; 3 Allergy Research Group, Institut de Recerca Vall d’Hebron, Universitat Autònoma de Barcelona, Barcelona, Spain; 4 Allergie-Centrum-Charité, Klinik für Dermatologie, Venerologie und Allergologie, Campus Charité Mitte, Universitätsmedizin Berlin, Charitéplatz 1, 10117 Berlin, Germany; 5 Division of Allergy and Immunology, Department of Internal Medicine, University of South Florida Morsani College of Medicine, Tampa, FL USA; 6 Allergy and Respiratory Research Group, Usher Institute of Population Health Sciences and Informatics, The University of Edinburgh, Edinburgh, UK; 7 Division of Allergy-Immunology, Department of Medicine, Northwestern University Feinberg School of Medicine, Chicago, IL USA; 8 Department of Allergy and Immunology, Hospital Quironsalud Bizkaia, Bizkaia, Spain; 9 Department of Allergy, Clinical Research Center for Allergy and Rheumatology, Sagamihara National Hospital, Kanagawa, Japan; 10 Pediatric Allergy & Immunology Unit, Children’s Hospital, Ain Shams University, Cairo, Egypt; 11 Department of Pediatric Allergy & Immunology, Emory University School of Medicine, Atlanta Allergy & Asthma, Atlanta, USA; 12 Division of Medicine, Academy of Medicine of Rio de Janeiro, Rio de Janeiro, Brazil; 13 Division of Allergy and Clinical Immunology, Department of Medicine, Quillen College of Medicine and Center for Excellence for Inflammation, Infectious Disease and Immunity, East Tennessee State University, Johnson City, TN USA; 14 Hospital Sírio Libanês, São Paulo, Brazil; 15 University Hospital of Montpellier, Montpellier, and Sorbonne Universités, Paris, France; 16 Department of Rheumatology, Allergy and Immunology, Tan Tock Seng Hospital, 11 Jalan Tan Tock Seng, Singapore; 17 Clínica El Avila, 6a.transversal Urb. Altamira, piso 8, consultorio 803, Caracas, 1060 Venezuela

**Keywords:** Aircraft, Air travel, Allergic reaction, Anaphylaxis, Asthma, Emergency, Flight, Food allergy

## Abstract

Allergic and hypersensitivity reactions such as anaphylaxis and asthma exacerbations may occur during air travel. Although the exact incidence of in-flight asthma and allergic emergencies is not known, we have concerns that this subject has not received the attention it warrants. There is a need to provide passengers at risk and airlines with the necessary measures to prevent and manage these emergencies. A review of the epidemiology, management and approaches to prevention of allergic and asthma emergencies during air travel is presented with the goal of increasing awareness about these important, potentially preventable medical events.

## Background

About 2.75 billion passengers are transported worldwide by airlines every year. Many of these people will have pre-existing long-term conditions – including asthma and/or allergy – which put them at risk of an in-flight medical emergency. In a smaller minority of people, these conditions may develop for the first time (i.e. incident cases) while in flight. It is expected that by 2030, half of all aircraft passengers will be over 50 years of age [1]. Up to 44,000 in-flight medical emergencies occur each year [2], and data suggest that about 17% of such cases are transferred to a hospital, with 4% resulting in hospitalization or death.

Systemic allergic reactions (SAR) (a serious systemic allergic reaction is defined as anaphylaxis) and asthma exacerbations (AE) may occur during air travel. We have concerns that to date, these have not received sufficient attention from passengers, airlines, and the medical community. In view of the potentially avoidable morbidity and mortality, a review about the epidemiology, management and prevention of allergic and asthma emergencies during air travel is needed to increase awareness about these reactions and how to prevent and treat them. Likewise, travel for the allergic and asthmatic passenger poses a number of problems, in particular for those with a history of a food-induced SAR or who have asthma. Patients may experience, among other, fear of suffering an allergic reaction or AE, problems regarding which medications to carry on board, how to use them, or not receiving appropriate attention when seeking support from airline personnel about their potential needs [3].

The frequency of these reactions among susceptible passengers is unknown; however, data from peanut allergic individuals indicate that approximately 9% of them have experienced some sort of allergic reaction during flights [4, 5].

## Medical emergencies during air travel

In-flight medical emergencies occur in 1 per 11,000 passengers [6] or 1 in every 604 flights [2]. A number of reasons are proposed to explain the incidence of medical and allergic events during air travel, and those are summarized in Table 1. The most common in-flight medical events include syncope, gastrointestinal and cardiac problems [2, 7, 8].

**Table 1:** Rick factors for medical and allergic events during air travel

An increase of passenger’s age	
Flight stress and anxiety, including increased security procedures	
Disruption of routine	
Changes in the cabin environment (temperature, humidity, air pressure)	
Decreased seat space	
Flight delays	
Alcohol/drug intake	
Longer flights	
Altered circadian rhythm	
Jet lag	
Pre-existing medical conditions	

## Alterations in passenger cabin during flight: implications of physiological changes during air travel to allergic and hypersensitivity diseases

There are two major concerns about allergic and respiratory diseases when traveling by air. The first is related to the concentration of oxygen in the cabin. While in flight, the cabin pressure is equivalent to an altitude of 6000 to 8000 ft, exposing passengers to a partial pressure of arterial oxygen of 60 mm Hg compared to 75 to 100 mm Hg at sea level. Thus, the partial pressure of oxygen in the cabin air at cruising altitude is 25–30% lower than at sea level. A slight fall in oxygen blood saturation occurs, ranging from 92 to 95%, followed by compensatory hyperventilation and tachycardia [9]. These physical alterations may affect passengers who have cardiac, respiratory or hematologic (anemia) diseases, theoretically increasing the risk of exacerbations of allergic and hypersensitivity reactions such as anaphylaxis.

These pressure changes can also result in “blocked ears or sinuses” or, occasionally, barotitis or barosinusitis. These conditions can usually be prevented by yawning, chewing gum during the flight, and by using a sympathomimetic nasal spray such as oxymetazoline before ascent and descent.

The dry cabin atmosphere (with humidity 6–18%) can also irritate the mucosal membranes of the mouth and upper respiratory tract. Likewise, dehydration can occur, especially in a passenger taking a diuretic. Therefore, drinking plenty of extra fluids is recommended during flight [10].

## Asthma and allergic reactions

Allergic reactions account for an estimated 2–4% of medical problems on board commercial airliners (Table 2) [5, 11–13]. Buehrle and Gabler observed that “allergy” was the 7th most common cause of in-flight medical problems between 2002 and 2007, ranging from 1.5 to 2.5% [1]. During the same period asthmatic events were 14th in frequency (0.6 to 3.2%).

**Table 2:** Prevalence of in-flight allergic reactions

Authors (year of publication)	Prevalence (%)	Reference
Szmajer et al. (2001) [13]	2.4	13
Delaune et al. (2003) [12]	2.8	12
Baltsezk (2008) [11]	3.7	11
Sand et al. (2012) [16]	2.2	17

Some authors propose that an AE is the most common, and potentially the most life-threatening condition reported by major airlines. According to Dowdall’s study, serious allergic reactions rarely occur. Respiratory events were the 5th cause of aircraft medical conditions, whereas allergic diseases, including urticaria, angioedema, additional forms of acute dermatitis, AE, and SAR were the 11th most common [6]. SAR in airplanes are most commonly triggered by foods (peanuts, tree nuts, and seafood) or medications. Very rarely these are triggered by insect stings or an insecticide spray [14].

There are reports of passengers having experienced idiopathic anaphylaxis while in flight, administering self-injected epinephrine (adrenaline) in the lavatory, and not notifying the flight attendant (Greenberger P, personal communication). There are also reports of attacks that have necessitated plane diversion to the closest airport.

Other publications report that allergic emergencies are responsible for 2.2% of all medical problems and result in 4.5% of aircraft diversions [2]. Allergic reactions were the 7th most frequent cause of medical events, and dermatologic manifestations, including skin rashes, the 9th most common cause in Baltszek’s study [11].

An article by Nable et al. on in-flight medical emergencies did not discuss allergic conditions or SAR, although it did mention a 12% incidence of “respiratory” reactions [8]. This prompted Casale and Lemanske to submit a letter to *The New England Journal of Medicine* highlighting the need to consider “anaphylaxis” as a reaction likely to occur during flights [15].

The lack of recognition of allergic reactions as an important cause of medical events during air travel could be due to the fact that they are rare, or because they are under-recognized, or diagnosed and not reported, since methods to report such reactions are different and not standardized [16, 17]. This is an area that therefore warrants further study. As previously stated, passengers often do not report medical problems during flight [5].

## In-flight treatment of allergic emergencies and asthma

Treating medical emergencies during flight is a major challenge and air travel is an important concern for subjects with asthma and a history of a SAR.

The resources to treat allergic emergencies are somewhat limited. In the United States, the Federal Aviation Administration requires the inclusion of epinephrine in medical kits carried on board [18]. These emergency medical kits typically contain the following medications [19]:

- Aqueous epinephrine (adrenaline) 1:10000 and 1:1000 dilution.
- Albuterol (salbutamol) for nebulization.
- Bronchodilator aerosol inhaler.
- Cortisol (hydrocortisone).
- Antihistamines tablets and injectable (commonly diphenhydramine).

A recommendation from this World Allergy Organization (WAO) expert group for in-flight treatment of a SAR and AE is:

- For AE, inhaled bronchodilator and oxygen. Consider an oral, intramuscular or intravenous corticosteroid for moderate to severe symptoms and intramuscular epinephrine for severe symptoms.
- For mild, moderate, and severe SAR, intramuscular epinephrine 0.01 mg/kg up to 0.5 mg of 1:1000 solution IM in the anterior lateral thigh. Repeat as necessary.

## Prevention

Food-induced SAR are increasingly being observed in many parts of the world. Strategies to reduce the risk of a SAR while traveling should begin during the early planning of the plane trip. Advice from their treating physician or allergist/immunologist should be obtained about preventive measures to be implemented before or during the flight. Likewise, a treatment plan should be instituted in case there is an inadvertent contact with a known allergen, i.e., a peanut [20–25].

Greenberger and Lieberman propose that subjects with idiopathic anaphylaxis not travel within a week of a previous episode and recommend the administration for an adult of prednisone 40–60 mg and an H1 antihistamine by mouth each morning for 1 week before travel. This can help reduce the frequency and severity of episodes [26]. If the flight is longer than 1 week after an episode of idiopathic anaphylaxis, empiric therapy should be initiated and continued to reduce the likelihood of an attack while in flight. For example, the passenger can be converted to alternate day prednisone.

Medical departments of major airlines can also be consulted about specific questions and recommendations for air flights on individual carriers. Physicians on board should be contacted immediately by the airline crew for any emergency, including allergic emergencies, to determine what treatment should be instituted and if the plane should be diverted to the nearest airport. In addition to medical kits on board and special training of the crew on the management of medical emergencies, airlines may have remote access to medical services which can monitor and give instructions for the best treatment until the plane arrives at a location where the passenger can be adequately treated.

Measures to avoid peanut and tree nut exposure for peanut and tree nut allergic individuals have resulted in lower odds of in-flight SAR from these foods (Table 3) [27, 28]. Since these measures may be difficult to implement, can cause discomfort for some passengers, and result in higher costs of travel, airlines and some travelers might be reluctant to implement them.

**Table 3:** Measures that reduce the risk of an in-flight reaction to peanut and tree nuts^a^

1. Passengers requesting any kind of special accommodation (e.g., peanut/tree nut snacks not be distributed, announcement to not eat items with peanut/tree nut, request special peanut/tree nut-free meal, buffer zone, pre-board, request to sit in a certain seat/zone).	
2. Peanut/tree nut-free meals.	
3. Wiping of tray tables	
4. Avoidance of airline pillows or blankets	
5. Buffer zones around which peanut or nut products cannot be consumed	
6. Request other passengers not to consume peanut/tree nut-containing products	
7. Announcement that passengers do not eat peanut/tree nut containing goods	
8. Not consuming airline-provided food	

^a^Modified from reference [28]

## Airline policies on allergy

Many airline carriers have devised strategies to prevent SAR and AE during travel (Table 4). However, there are no standardized measures to do so and each carrier has its own recommendations. Retrieving first-hand information about SAR and AE is difficult [29]. A summary of proposed airline policies is presented in Table 5 [30].

**Table 4:** Airline policies for allergic passengers (Data from 13 air carriers) ^a^

	Number of airlines
Pre-boarding arrangements	
• Request a food “buffer zone”	5
• No “buffer zone”	3
• Request “allergen-free meal”	1
• Pre-boarding to wipe down seats and table trays	Yes 4
	No 1
• Buffer zone for fragrance sensitivity	2
• Announcements to inform customers there is a peanut or tree-nut allergic passenger on board or that peanut products will not be served	Yes 4
	No 7
Free snack policy on peanut/nut/sesame	
• Serving peanuts	Yes 2
	No 11
• Serving nuts and sesameaw	Yes 8
	No 1
• First class warmed nuts	3
Gluten-free meals	Yes 11
	No 1
Lactose-free meals	1
Shellfish served	Yes 4
	No 3
Fish served	Yes 6
	No 1
Buffer zones for passengers allergic to pets	5

^a^ In general air lines cannot guarantee an entirely peanut-free environment. Modified from reference [30]

**Table 5:** Recommendations to prevent and manage in-flight allergic events

• Promote the prevention of allergic diseases via passenger education • Medical consultation for high-risk passengers before traveling • Train and re-train aircrews • Promote general preventive measures during the flight: hydration, food allergen avoidance (especially peanuts, tree nuts, other foods, as necessary) • Provide an appropriate place for furry pets away from subjects with pet allergy • Provide for sufficient quantities of appropriate medications: epinephrine (adrenaline), β2 agonists for inhalation and nebulization, oral and injectable corticosteroids and antihistamines • Oxygen	

## Conclusions

As greater numbers of people fly, the number of AE and SAR are likely to increase during flights in the future. Therefore, passengers at risk should be aware of the necessary measures to prevent and manage these emergencies. It is also vitally important that airlines are prepared to deal with these diseases by providing the necessary strategies to decrease the incidence of SAR and AE. They also should have the necessary means to treat these reactions when and if they occur. There is a clear opportunity for airlines to work alongside allergists/immunologists to implement evidence-based recommendations to prevent allergic reactions during flight, especially SAR.

## Abbreviations

AE: Asthma exacerbation
SAR: Systemic allergic reaction
WAO: World Allergy Organization
